# Revisiting egg yolk involvement in children’s food allergy to egg

**DOI:** 10.1186/2045-7022-3-S3-P86

**Published:** 2013-07-25

**Authors:** C Brossard, F Rancé, A Juchet, M Drouet, E Paty, S Legoué-Morillon, F Nau, M Anton, S Denery

**Affiliations:** 1 UR1268 Biopolymères, Interactions, Assemblages, INRA, Nantes, France; 2 Pédiatrie - Pneumologie, Allergologie, CHU Toulouse, Toulouse, France; 3 Pneumologie - Unité Allergologie Générale, CHU d'Angers, Angers, France; 4 Pneumo Allergologie Infantile, CHU Necker, Paris, France; 5 Centre de Recherche et d'Education en Allergie Alimentaire, CHU de Nantes, Nantes, France; 6 UMR1253 Sciences et Technologie du Lait et de l'Oeuf, INRA/AgroCampus Ouest, Rennes, France

## Background

Food allergy to egg is commonly diagnosed in childhood with good prognosis. Egg white is considered as the main culprit with major 4 identified allergens (Gal d 1 to Gal d 4). Egg yolk (with two identified allergens Gal d 5 and Gal d 6 in the livetin fraction) retained less attention and its clinical involvement remains unclear. This study revisits sensitization to both egg fractions in a cohort of children allergic to egg and investigates IgE reactivity toward the main egg yolk fractions.

## Methods

Children with food allergy to egg were recruited. Crude egg and crude white and yolk fractions, carefully prepared to avoid any contamination between yolk and white, were used for prick-tests in this cohort. Specific IgE in sera towards egg yolk fractions (lipovitellins/HDL, lipovitellenins/LDL, phosvitins and livetins) were determined by ELISA.

## Results

Fifty-two children (13 girls; 3.9 ± 1.7 years old) with food allergy to egg were recruited. They mainly suffered from urticaria (48%) and to a lesser extend from eczema (11%), rhinitis (11%) and gastrointestinal troubles (9%). At inclusion, all children had positive prick-tests to crude egg and crude white fraction, 78% to crude yolk fraction. Diameters of positive prick-tests were 10.1 ± 5.2 mm for crude egg; 12.7 ± 7.2mm for egg white and 8.9 ± 5.1 mm for egg yolk. Among egg yolk fractions, IgE in sera interacted more frequently with lipovitellenins/LDL (30%) and livetins (30%) than lipovitellins/HDL (16%) and phosvitins (4%) fractions.

## Conclusion

Beside sensitization to egg white, children with allergy to egg appear to be more frequently sensitized to egg yolk than generally assumed. IgE reactivity to egg yolk is more directed toward the plasma proteins in lipovitellenins/LDL and livetins fractions than the granules proteins in lipovitellins/HDL and phosvitins fractions.

## Disclosure of interest

None declared.

